# Dual pH-sensitive and UCST-type thermosensitive dendrimers: phenylalanine-modified polyamidoamine dendrimers with carboxyl termini†

**DOI:** 10.1039/c8ra05381b

**Published:** 2018-08-06

**Authors:** Mamiko Tamaki, Daichi Fukushima, Chie Kojima

**Affiliations:** Department of Applied Chemistry, Graduate School of Engineering, Osaka Prefecture University 1-1 Gakuen-cho, Naka-ku Sakai Osaka 599-8531 Japan kojima@chem.osakafu-u.ac.jp +81 72 254 8190 +81 72 254 8190

## Abstract

Dendrimers are unique polymers with well-defined structures, and are useful as functional unimolecular nanoparticles. Previous reports have shown that polyamidoamine (PAMAM) dendrimers modified with hydrophobic molecules, such as amino-terminal phenylalanine (Phe), are thermosensitive at high pH. In the present study, we designed carboxyl-terminal Phe-modified PAMAM dendrimers that are thermosensitive under acidic conditions. We reacted an amino-terminal PAMAM dendrimer with various acid anhydrides, such as succinic anhydride, cyclohexanedicarboxylic anhydride, and phthalic anhydride, prior to the reaction with Phe. Interestingly, the amino-terminal Phe-modified PAMAM dendrimers exhibited LCST (lower critical solution temperature)-type thermosensitivity at approximately pH 7, but the carboxyl-terminal Phe-modified dendrimers exhibited UCST (upper critical solution temperature)-type thermosensitivity in acidic solutions. Temperature sensitivity was dependent on both pH and the anhydride modifier. We were able to separate rose bengal (a model compound) from aqueous solutions of the carboxyl-terminal Phe-modified dendrimer at low pH.

## Abstract 2

We designed carboxyl-terminal Phe-modified PAMAM dendrimers by using various acid anhydrides. These dendrimers exhibited UCST-type thermosensitivity, which was dependent on both pH and the anhydride modifier.

## Graphical abstract

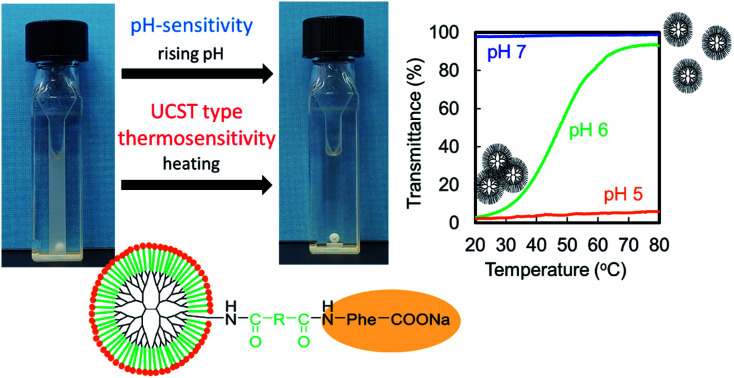

There are many reports of smart materials that are capable of responding to some stimuli such as temperature, pH, or light.^1–3^ pH-responsive polymers can be designed by adding ionic groups to polymers. Polymers comprising weak acids such as poly(methacrylic acid) respond at low pH, and polymers consisting of weak bases such as poly[(2-dimethylamino)ethyl methacrylate] respond at high pH.^2^ Thermosensitive (temperature-responsive) polymers undergo dramatic changes in solubility at the phase transition temperature. Such polymers can be applied to green chemistry and biomedical applications, *e.g.*, chromatography, drug delivery systems, gene therapy, thermally switchable optical devices, and separation of substances.^1,3^ Thermosensitive polymers can be divided into two types: lower critical solution temperature (LCST)-type polymers and upper critical solution temperature (UCST)-type polymers.^4,5^ There have been many reports on LCST-type thermosensitive polymers such as poly(*N*-isopropyl acrylamide) (PNIPAm).^4,5^ Studies on UCST-type thermosensitive polymers, however, are much less common, although poly(*N*-acryloyl glycinamide) (PNAGA), ureido polymers and poly(sulfobetaine)s have been reported.^4,5^ Dual stimuli-responsive polymers have been developed to construct more sophisticated smart systems. Because pH and temperature are easily controlled, dual pH-sensitive and thermosensitive materials are receiving considerable attention.^6,7^

Dendrimers are polymers with a unique branched structure. They are synthesized in step-wise reactions, so their molecular weights, shapes, and sizes can be precisely defined. Furthermore, dendrimers can encapsulate and/or modify various kinds of molecules. Many researchers have used dendrimers as nanocarriers or platforms for smart materials, and there are many review articles about dendrimers.^8–14^ Among a number of nanocarrier, such as dendrimers, liposomes, polymeric micelles and linear polymers, much attention has been paid to dendrimers because of their advantages for biomedical applications. It is because dendrimers have the ability to maintain drug levels in a therapeutically desirable range, the capability to deliver a variety of bioactive molecules and targeting ability. Dendrimers also increase half-life, solubility, stability, and permeability of drugs, improve delivery efficiency, and reduce macrophage uptake and side effects.^14^ Polyamidoamine (PAMAM) dendrimers were one of classical dendrimers, and have been widely studied.^8,9^ In previous studies, PAMAM dendrimers modified with various hydrophobic acids, oligo-(ethylene glycols), and an *N*-isopropyl group exhibited LCST-type thermosensitivity.^12,15–18^ LCST-type thermosensitive dendrimers have also been prepared by attaching hydrophobic amino acids such as phenylalanine (Phe) and/or leucine (Leu) to PAMAM polymers.^19^ Such PAMAM dendrimers also respond to pH owing to their terminal and inner tertiary amines. However, they are not thermosensitive in acidic solutions because these amines are protonated and become much hydrophilic. pH-sensitive polymers that respond between acid and neutral conditions are more useful for biomedical applications. It is because the physiological pH is 7.4 and the pH in various internal organs and organelles is lower: stomach (pH 1–3), lysosomes (pH 4.5–5), endosomes (pH 5–6.5), and tumor (pH 6.5–7.2).^3^

In the present study, we designed Phe-modified PAMAM dendrimers with carboxyl termini that were thermosensitive in acidic solutions. We reacted amino-terminal PAMAM dendrimers with acid anhydrides prior to the reaction with Phe. We used succinic anhydride (Suc), cyclohexanedicarboxylic anhydride (CHex), and phthalic anhydride (Ph) to compare thermosensitivity and pH-sensitivity in the dendrimers. Finally, we demonstrated the recovery of a useful compound (rose bengal, a model compound) from aqueous solution using a carboxyl-terminal Phe-Ph-modified dendrimer.

The carboxyl-terminal Phe-modified dendrimers were synthesized according to Fig. 1. First, we reacted a fourth-generation (G4) amine-terminal PAMAM dendrimer with each of the three different acid anhydrides (Suc, CHex, and Ph) to produce carboxyl-terminal dendrimers. We then reacted the carboxyl groups of the PAMAM dendrimers with carboxyl group-protected Phe. Phenylalanine benzyl ester (Phe-OBzl) was used for the Suc- and CHex-reacted dendrimers. Phenylalanine methyl ester (Phe-OMe) was used for the Ph-reacted dendrimer, owing to the reduced steric hindrance. After removing the protecting groups from Phe in an alkaline solution, we obtained G4-Suc-Phe, G4-CHex-Phe, and G4-Ph-Phe. The numbers of acid anhydride and Phe residues bound to the dendrimers were estimated from the proton nuclear magnetic resonance (^1^H NMR) spectra (Fig. S1†). In the spectrum of G4-Suc-Phe (Fig. S1A†), the bound numbers of Phe and Suc to the dendrimer were evaluated from the integral ratios of the signals at 4.3 ppm (Phe) and 2.2 ppm (Suc) to 2.6 ppm (dendrimer), respectively. Because the signal at 2.2 ppm for Suc was overlapped with the dendrimer signal, the integral ratio for Suc was obtained by subtracting the dendrimer signal. In the spectrum of G4-CHex-Phe (Fig. S1B†), the bound numbers of Phe and CHex to the dendrimer were evaluated from the integral ratios of the signals at 4.2–4.3 ppm (Phe) and 1.1–1.9 ppm (CHex) to 2.2 ppm (dendrimer), respectively. In the spectrum of G4-Ph-Phe (Fig. S1C†), the bound numbers of Phe and Ph to the dendrimer were evaluated from the integral ratios of the signals at 4.4 ppm (Phe) and 7.0–7.4 ppm (Ph) to 2.2 ppm (dendrimer), respectively. Because the signal at 7.0–7.4 ppm for Ph was overlapped with the Phe signal, the integral ratio for Ph was obtained by subtracting the Phe signal. The bound numbers of acid anhydride and Phe to the dendrimer were listed in Table S1.† These shows that almost all the termini of the dendrimers were modified with both the acid anhydride and Phe.

**Fig. 1:**
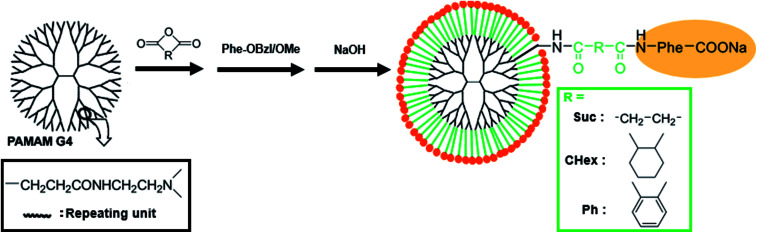
Preparation of PAMAM dendrimers modified with Phe *via* linkers.

We investigated the temperature-dependent transmittance of synthesized dendrimers at various pH values (Fig. 2). The G4-Suc-Phe solution was clear at pH 6, but turbid at pH 4. The pH sensitivity is caused by the protonation behavior of the inner tertiary amines and terminal carboxyl groups. The dendrimer was highly soluble at pH 6 owing to the deprotonated terminal carboxyl groups. However, it is likely that the carboxyl group became protonated and the negative charge disappeared at pH 4. At pH 5, the dendrimer exhibited UCST-type thermosensitivity. G4-CHex-Phe and G4-Ph-Phe behaved in a similar way to G4-Suc-Phe: solutions of these polymers were clear at high pH and turbid at low pH, and the dendrimers exhibited UCST-type thermosensitivity at the intermediate pH. Carboxyl-terminal dendrimers without Phe, such as G4-Suc, G4-CHex, and G4-Ph, did not exhibit UCST-type thermosensitivity, even at low pH (Fig. S2†). Therefore, the Phe modification induced UCST-type thermosensitivity. These results indicate that the carboxyl-terminal Phe-modified dendrimers were dual pH-sensitive and UCST-type thermosensitive materials.

**Fig. 2:**
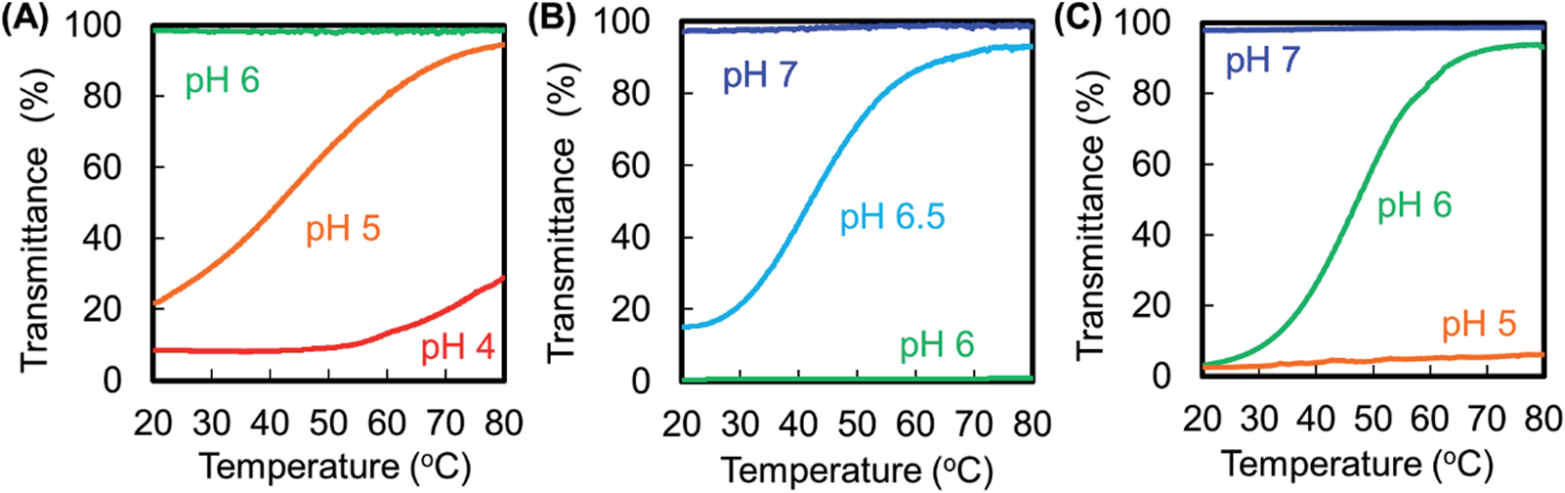
Temperature-dependent transmittance curves of (A) G4-Suc-Phe, (B) G4-CHex-Phe, and (C) G4-Ph-Phe at various pH values.

The phase transition temperature was defined as the temperature at which the light transmittance was 50%. Table 1 summarizes the thermosensitivity of the Phe-modified PAMAM dendrimers. The amino-terminal Phe-modified dendrimers (G4-Phe-NH_2_) exhibited LCST-type thermosensitivity at basic pH, but not at acidic pH, as shown in Fig. S3.†^19^ However, the carboxyl-terminal Phe-modified dendrimers had UCST-type thermosensitivity at acidic pH. Because PAMAM dendrimers have numerous inner tertiary amines, amino-terminal Phe-modified dendrimers are classified as polycationic polymers. However, carboxyl-terminal Phe-modified dendrimers can become zwitterionic, when the pH is adjusted. Some zwitterionic polymers exhibit UCST-type thermosensitivity.^4,5^ It is likely that the UCST-type thermosensitivity of carboxyl-terminal Phe-modified dendrimers is based on the zwitterionic model. The carboxyl-terminal Phe-modified dendrimers aggregated at low temperature, owing to the interaction between the terminal carboxyl anions of Phe and the ammonium cations of PAMAM, which were dissociated by heating to induce UCST-type thermosensitivity. Therefore, we think that the type of thermosensitivity of the amino- and carboxyl-terminal Phe-modified dendrimers depends on the difference in the ionic state. To investigate the UCST-type phase transition mechanism, we examined the dendrimer solutions with the UCST-type thermosensitivity by using an optical microscope. Spherical droplets were observed at low temperature, and they decreased with increasing temperature (Fig. 3). This suggests that coacervation, that is liquid–liquid phase separation, occurred at low temperature in our dendrimers. Previously, ureido polymers exhibited UCST-type thermosensitivity, which resulted from a coacervate formation. These ureido polymers showed the salt sensitivity, at which the phase transition temperature showed a rise of more than 10 °C in the presence of salt.^20^ The phase transition behavior of our dendrimer was compared in the absence and presence of 150 mM NaCl. The temperature-dependent transmittance curves were almost similar, although the phase transition temperature slightly increased from 47 °C to 53 °C (Fig. S4†). Thus, the salt effect was limited in our dendrimer. The detailed phase transition mechanisms remain to be investigated.

**Table 1:** Thermoresponsive behavior of various dendrimers modified with Phe *via* linkers

Dendrimer	Turbid	UCST	Clear	LCST
G4-Suc-Phe	pH 4	pH 5 (42 °C)	pH 6	N.D.
G4-CHex-Phe	pH 6	pH 6.5 (42 °C)	pH 7	N.D.
G4-Ph-Phe	pH 5	pH 6 (47 °C)	pH 7	N.D.
G4-Phe-NH_2_	N.D.	N.D.	pH 5	pH 7.4 (34 °C)

**Fig. 3:**
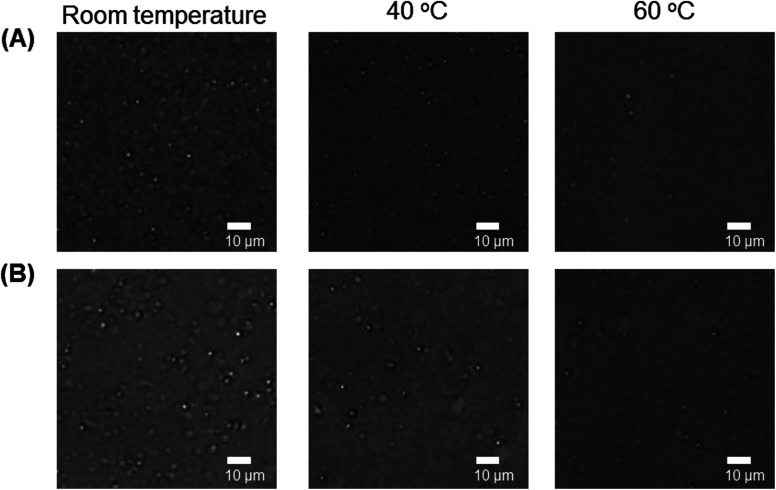
Microscopic images of dendrimer solutions at different temperatures. (A) G4-Suc-Phe at pH 5 and (B) G4-Ph-Phe at pH 6.

Next, we compared sensitivity to various stimuli in dendrimers with different linkers. Thermosensitivity was similar among these dendrimers, but pH sensitivity differed. We observed UCST-type thermosensitivity in the more hydrophobic dendrimers at higher pH. The pH values in G4-CHex-Phe and G4-Ph-Phe were higher than in G4-Suc-Phe. The partition coefficients (log *P* values) of the acid anhydrides were calculated as −0.36, 1.11, and 1.43 for Suc, CHex, and Ph, respectively, based on Crippen's fragmentation model.^21^ This suggests that Suc is more hydrophilic than the other anhydrides. Stimuli-sensitivity can be controlled by changing the acid anhydride linker. The bound amino acid may also affect pH sensitivity and thermosensitivity, as we have reported previously.^19^ Stimuli-responsive properties can be tuned for specific applications.

Finally, we demonstrated the usefulness of our dual stimuli-sensitive dendrimer in an application. PAMAM dendrimers can be used to encapsulate anionic compounds such as rose bengal (RB),^22–24^ and are therefore useful for the separation. We mixed RB and G4-Ph-Phe at a ratio of 15/1 in the isotonic solutions at pH 5 or 7. After centrifugation, the absorbance of the mixture was compared with that of the free RB solution. G4-Ph was used instead of G4-Ph-Phe as a control. The absorption spectra of free RB, and mixtures of RB and G4-Ph or RB and G4-Ph-Phe were almost the same at pH 7. However, absorption decreased dramatically in the mixture of RB and G4-Ph-Phe at pH 5 after centrifugation (Fig. 4). The residual (%) was calculated from the ratio of the absorbance of the RB solution in the presence of the dendrimer to the absorbance of the RB solution in the absence of the dendrimer after centrifugation. Almost all the RB molecules were retained in the presence of our dendrimers at pH 7, but residual RB decreased to 73% and 9% at pH 5 in the presence of G4-Ph and G4-Ph-Phe, respectively. Because G4-Ph-Phe is insoluble at pH 5, the RB molecules in the solution were encapsulated and condensed into the dendrimer droplets. However, the RB molecules encapsulated in G4-Ph were not condensed effectively because G4-Ph is mostly soluble at pH 5 (Fig. S5†). Both dendrimers were soluble at pH 7, and it was impossible to separate the RB molecules from the solution by centrifugation (Fig. 2 and S5†). Our results suggest that some compounds can be separated from aqueous solution by changing the solubility of carboxyl-terminal Phe-modified dendrimers.

**Fig. 4:**
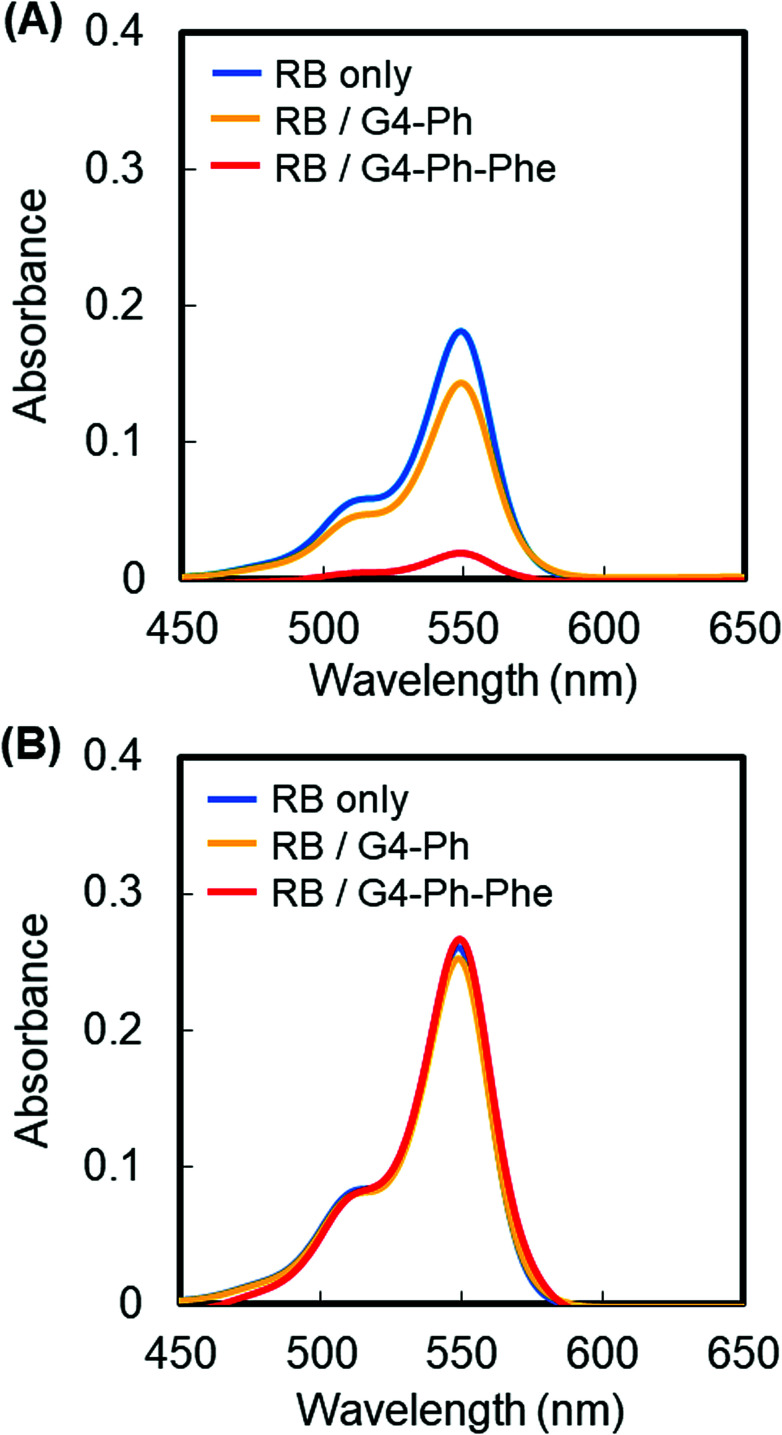
Absorption spectra of RB ([RB] = 4 μM) in the absence and presence of G4-Ph and G4-Ph-Phe after centrifugation at pH 5 (A) and pH 7 (B).

In conclusion, we synthesized three different carboxyl-terminal Phe-modified PAMAM dendrimers using three different acid anhydrides as linkers. Each dendrimer exhibited both pH sensitivity and UCST-type thermosensitivity, which were dependent on the hydrophobicity of the linker. The dendrimers were capable of encapsulating RB (a model compound), and it was possible to separate RB from an aqueous solution by decreasing the pH. This indicates that the dual stimuli-sensitive dendrimers (sensitive to both pH and temperature) are useful for separation of substances. To the best of our knowledge, this is the first report of UCST-type dendrimers. The detailed characterization of this kind of dendrimer is ongoing.

## Conflicts of interest

There are no conflicts of interest to declare.

## Supplementary Material

RA-008-C8RA05381B-s001
