# Supplementary material for: Dual pH-sensitive and UCST-type thermosensitive dendrimers: phenylalanine-modified polyamidoamine dendrimers with carboxyl termini
Source: RSC Adv. 2018 Aug 6;8(49):28147–51. doi: 10.1039/c8ra05381b (PMC9084302; doi:10.1039/c8ra05381b)
Supplement: RA-008-C8RA05381B-s001 [file RA-008-C8RA05381B-s001.pdf]

Electronic Supplementary Material (ESI) for RSC Advances.
This journal is © The Royal Society of Chemistry 2018

Supporting Information

Dual pH-Sensitive and UCST-Type Thermosensitive Dendrimers: Phenylalanine-Modified Polyamidoamine Dendrimers with Carboxyl Termini

Mamiko Tamaki[1], Daichi Fukushima[1], Chie Kojima[1]*

Experimental procedures

Synthesis of carboxyl-terminal dendrimers

A fourth-generation (G4) PAMAM dendrimer with an ethylenediamine core was purchased from Aldrich Chemical Company (Milwaukee, WI). The PAMAM dendrimer (56 mg, 3.9 μmol) was dissolved in 125 mM $NaHCO_3$ aqueous solution (5.5 mL). An excess of acid anhydride (Suc or CHex, approximately 100 eq) was added to the dendrimer solution. Each mixed solution was stirred overnight at room temperature, then the solution pH was adjusted to approximately 10 using 4 M aqueous NaOH. The carboxyl-terminal dendrimers were purified by dialysis (molecular weight cut-of (MWCO): 1000) in distilled water, and white solids were obtained after lyophilization. The yields of G4-Suc and G4-CHex were 95 mg (110%) and 62 mg (64%), respectively. ^1H NMR of G4-Suc (D_2O containing NaOD): 2.24-2.28 (br, $NCH_2C\underline{H_2}CONHCH_2CH_2N$ for inner and terminal chains of PAMAM and methylene for Suc), 2.46 (br, $NCH_2CH_2CONHCH_2C\underline{H_2}N$ for inner chains of PAMAM), 2.65 (br, $NC\underline{H_2}CH_2CONHCH_2CH_2N$ for inner and terminal chains of PAMAM), and 3.13 (br, $NCH_2CH_2CONHC\underline{H_2}CH_2N$ for inner and terminal chains of PAMAM and $NCH_2CH_2CONHCH_2C\underline{H_2}N$ for terminal chains of PAMAM). ^1H NMR of G4-CHex (D_2Ocontaining NaOD): 1.21-1.83 (m, methylene for CHex), 2.26 (br, $NCH_2C\underline{H_2}CONHCH_2CH_2N$ for PAMAM), 2.41-2.55 (br, $NCH_2CH_2CONHCH_2C\underline{H_2}N$ for inner chains of PAMAM and methine for CHex), 2.66 (br, $NC\underline{H_2}CH_2CONHCH_2CH_2N$ for inner and terminal chains of PAMAM), and 3.14 (br,

$NCH_2CH_2CONHC\underline{H_2}CH_2N$ for inner and terminal chains of PAMAM and $NCH_2CH_2CONHCH_2C\underline{H_2}N$ for terminal chains of PAMAM).

The G4 PAMAM dendrimer (57 mg, 4.0 μmol) was dissolved in a dimethyl sulfoxide (DMSO)/N,N-dimethyl formamide (DMF) mixture (2.4 mL, 49/11). Excess phthalic anhydride (0.31 g, 2.1 mmol) and triethylamine (TEA, 200 μL, 1.44 mmol) were added to the dendrimer solution. After stirring overnight at room temperature, the dendrimer was purified by dialysis (MWCO: 1000) with DMSO and distilled water. White solid was obtained after lyophilization. Yield: 67 mg (63 %). ^1H NMR of G4-Ph (D_2O containing NaOD): 2.19 (br, $NCH_2C\underline{H_2}CONHCH_2CH_2N$ for inner and terminal chains of PAMAM), 2.36 (br, $NCH_2CH_2CONHCH_2C\underline{H_2}N$ for inner chains of PAMAM), 2.57 (br, $NC\underline{H_2}CH_2CONHCH_2CH_2N$ for inner and terminal chains of PAMAM), 3.02-3.24 (br, $NCH_2CH_2CONHC\underline{H_2}CH_2N$ for inner and terminal chains of PAMAM and $NCH_2CH_2CONHCH_2C\underline{H_2}N$ for terminal chains of PAMAM), and 7.17-7.41 (m, phenyl for Ph).

Reaction of carboxyl-terminal dendrimers with carboxyl groups-protected Phe

G4-Suc (83 mg, 3.8 μmol) was dispersed in DMSO (5 mL) and stirred overnight. After stirring, 3-phenyl-L-alanine benzyl ester 4-toluene sulfonate (Phe-OBzl·Tos, 0.12 g, 0.30 mmol), 1-[bis(dimethylamino)methyliumyl]-1*H*-benzotriazole-3-oxide hexafluorophosphate (HBTU, 0.11 g, 0.28 mmol), and triethylamine (TEA, 41 μL, 0.29 mmol) were added to the dendrimer solution and stirred at room temperature. After stirring for 4 days, 1 mL of distilled water was added. The dendrimer was purified by dialysis (MWCO: 1000) in methanol. G4-Suc-Phe-OBzl was obtained following the evaporation of methanol and lyophilization. The yield was 88 mg (66%). ^1H NMR (MeOD containing DCl): 2.33 (s, CH_3 for tosyl), 2.41-2.49 (methylene for Suc), 2.70-3.04(m, $NCH_2C\underline{H_2}CONHCH_2CH_2N$ for inner and terminal chains of PAMAM and $NCH_2CH_2CONHCH_2C\underline{H_2}N$ for inner chains of PAMAM and H_β for Phe), 3.25-3.62 (br, $NC\underline{H_2}CH_2CONHCH_2CH_2N$ for inner and terminal chains of PAMAM and $NCH_2CH_2CONHC\underline{H_2}CH_2N$ for inner and terminal chains of PAMAM and $NCH_2CH_2CONHCH_2C\underline{H_2}N$ for terminal chains of PAMAM), 4.62 (br, H_α for Phe), 5.02 (br, benzyl

for Phe), 7.11-7.26 (m, phenyl for Phe and tosyl), and 7.69 (m, phenyl for tosyl).

G4-CHex-Phe-OBzl was synthesized by reacting G4-CHex (53 mg, 2.11 μmol) with Phe-OBzl·Tos (0.12 g, 0.30 mmol), HBTU (0.09 g, 0.24 mmol) and TEA (41 μL, 0.29 mmol) in 5 mL of DMSO.The yield was 84 mg (109 %). ^{1}H NMR (MeOD containing DCl): 1.27-2.03 (m,methylene for CHex), 2.34 (s, $C\mathit{H}_3$ for tosyl), 2.50-3.12(m, $NCH_2C\underline{H_2}CONHCH_2CH_2N$ for inner and terminal chains of PAMAM and $NCH_2CH_2CONHCH_2C\underline{H_2}N$ for inner chains of PAMAM and H_β for Phe), 3.44-3.58 (br, $NC\underline{H_2}CH_2CONHCH_2CH_2N$ for inner and terminal chains of PAMAM and $NCH_2CH_2CONHC\underline{H_2}CH_2N$ for inner and terminal chains of PAMAM and $NCH_2CH_2CONHCH_2C\underline{H_2}N$ for terminal chains of PAMAM and methine for CHex), 4.56-4.65 (br, H_α for Phe), 5.00 (br, benzyl for Phe), 7.15-7.28 (m, phenyl for Phe and tosyl), and 7.67-7.69 (m, phenyl for tosyl).

G4-Ph-Phe-OMe was synthesized by reacting G4-Ph (57 mg, 2.2 μmol) with Phe-OMe·HCl (0.12 g, 0.28 mmol), HBTU (0.11 g, 0.290 mmol) and TEA (41 μL, 0.29 mmol) in 3 mL of DMSO. The yield was 77 mg (109 %). ^{1}H NMR (MeOD containing DCl): 2.68-2.73 (m, $NCH_2C\underline{H_2}CONHCH_2CH_2N$ for inner and terminal chains of PAMAM and $NCH_2CH_2CONHCH_2C\underline{H_2}N$ for inner chains of PAMAM and H_β for Phe), 3.33 (s, $OC\mathit{H}_3$), 3.10-3.78 (br, $NC\underline{H_2}CH_2CONHCH_2CH_2N$ for inner and terminal chains of PAMAM and $NCH_2CH_2CONHC\underline{H_2}CH_2N$ for inner and terminal chains of PAMAM and $NCH_2CH_2CONHCH_2C\underline{H_2}N$ for terminal chains of PAMAM), 4.75 (br, H_α for Phe), and 7.19-7.76 (m, phenyl for Ph and Phe).

Deprotection of Phe at the dendrimers' periphery

G4-Suc-Phe-OBzl (101 mg, 2.9 μmol), G4-CHex-Phe-OBzl (84mg, 2.2 μmol) and G4-Ph-Phe-OMe (77 mg, 2.4 μmol) were dissolved in methanol (4 mL). 4 M NaOH methanol solution (500 μL) was added. After stirring at 4°C for 2 h, these dendrimers were dialyzed (MWCO: 1000) in distilled water. White solids were obtained. Yields of G4-Suc-Phe, G4-CHex-Phe and G4-Ph-Phe were 57 mg (64 %),

55 mg (77 %), and 63 mg (81 %), respectively.

Characterization

The ^{1}H NMR spectra were obtained using a ^{1}H-NMR spectrometer (JEOL, 400 MHz) (JEOL Ltd., Tokyo, Japan). LogP values were calculated by Crippen's fragmentation, using ChemBioDraw Ultra 13.0 software.

The temperature-dependent transmittance at 500 nm was measured using a Jasco Model V-630 UV/Vis spectrophotometer equipped with an ETC-717 cell holder (Jasco Inc., Tokyo, Japan). The heating rate was 1.0°C/min. The dendrimer solutions (1 mg/mL, 20 mM buffer) were prepared, and the pH was adjusted to 6 or higher using phosphate buffer, and to pH 4 or 5 using acetate buffer.

Microscopic observation was performed by using inverted fluorescence microscopy (ECLIPSE Ti-U, Nikon Corp. (Tokyo, Japan)) equipped with a glass heater unit for cell culture (C-140A, BLAST Inc., Kanagawa, Japan). 50 μL of dendrimer solutions (1 mg/mL) were dropped on a slide glass, which were observed at room temperature, 40 °C and 60 °C.

Separation of rose bengal from aqueous solutions

RB (4 nmol) and G4-Ph-Phe (0.27 nmol) were mixed at a ratio of 15/1 in 1 mL of 20 mM acetate buffer (pH 5) or 20 mM phosphate buffer (pH 7) solution containing 150 mM NaCl. The mixed solutions were incubated at room temperature for 20 min. After centrifugation (15000 g, 5 min) at 25 ^{o}C, the absorption spectra of the mixture were measured by the Jasco Model V-630 UV/Vis spectrophotometer to compare with those of the free RB solution. G4-Ph was used instead of G4-Ph-Phe to do the same experiment. The residual RB (%) was calculated from the ratio of the absorbance of the RB and dendrimer mixed solution after the centrifugation to the absorbance of the free RB solution after the centrifugation. The absorbance at the wavelength of the peak top in the free RB solution was compared.

Table S1. List of dendrimers synthesized in the present study.

dendrimer	terminal number	bound number	
		acid anhydride	Phe
G4-Suc-Phe	64	64 (Suc)	57
G4-CHex-Phe	64	58 (CHex)	51
G4-Ph-Phe	64	71 (Ph)	48

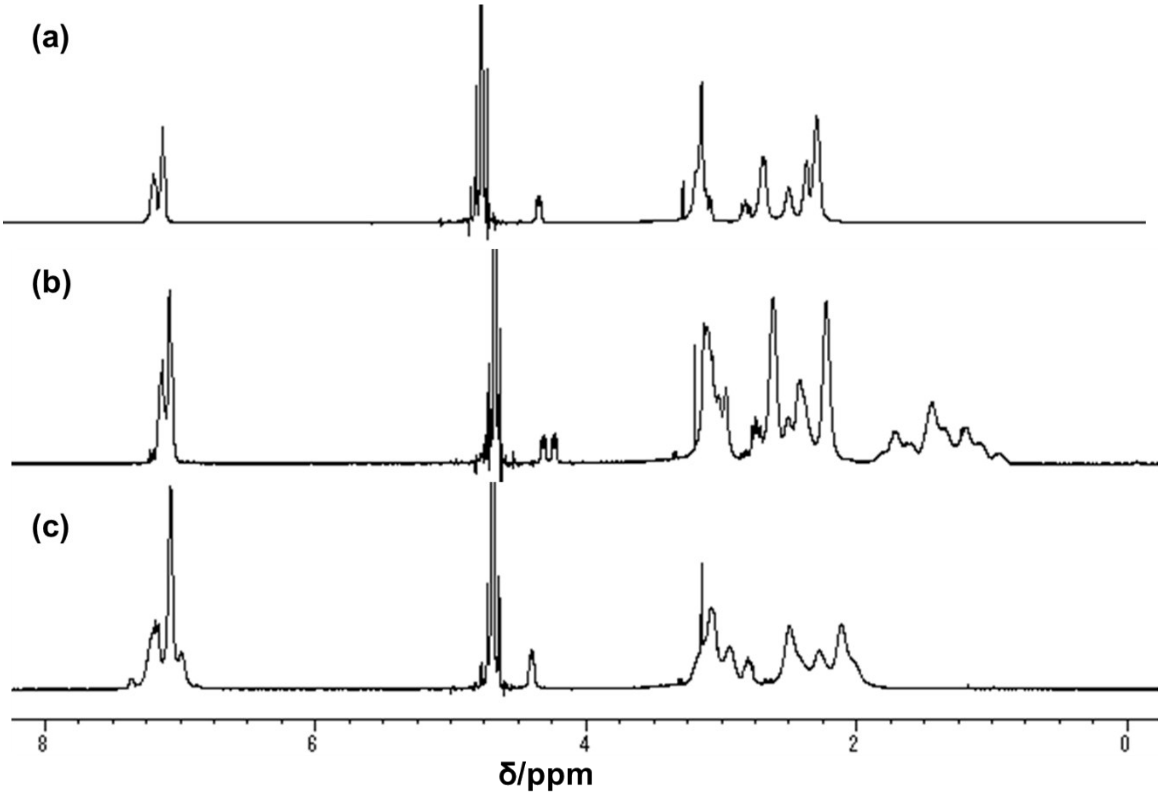

Figure S1. ^{1}H NMR spectra of (a) G4-Suc-Phe, (b) G4-CHex-Phe, and (c) G4-Ph-Phe in D_2O containing NaOD.

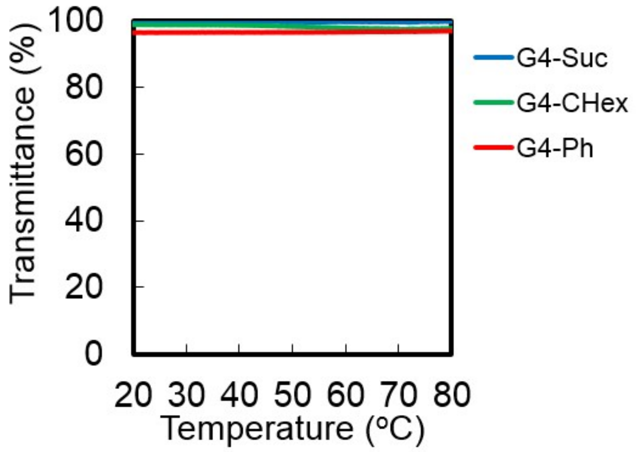

Figure S2. Temperature-dependent transmittance curves of G4-Suc, G4-CHex and G4-Ph at pH 4.

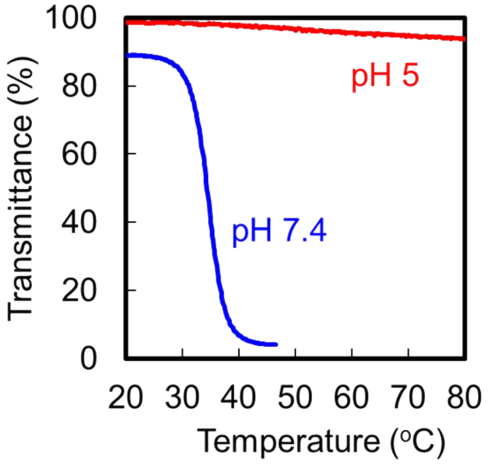

Figure S3. Temperature-dependent light transmittance curves of G4-Phe-NH_2 at pH 7.4 and pH 5.

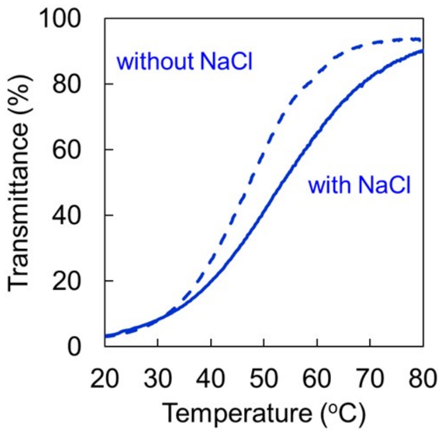

Figure S4. Temperature-dependent transmittance curves of G4-Ph-Phe at pH 6 in the absence and presence of 150 mM NaCl.

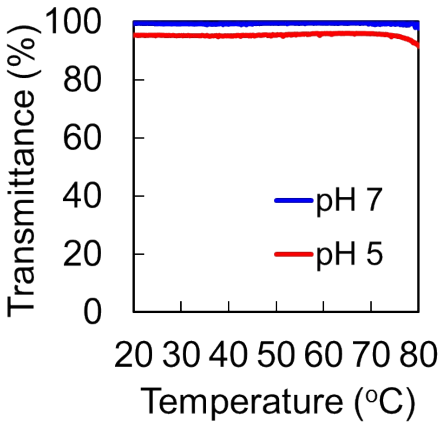

Figure S5. Temperature-dependent transmittance curves of G4-Ph at pH 5 and pH 7.
